# Clara Southmayd Ludlow: Her Thirst for Knowledge was Positively Inspirational: Honoring a Female Giant in Tropical Medicine

**DOI:** 10.4269/ajtmh.17-ludlow

**Published:** 2017-12-06

**Authors:** Stephen Higgs, Patricia F. Walker, Karen A. Goraleski, Authors are members of the American Society of Tropical Medicine and Hygiene’s Executive Committee

**Affiliations:** 1 Immediate Past President;; 2 President;; 3 Executive Director

**Figure:**
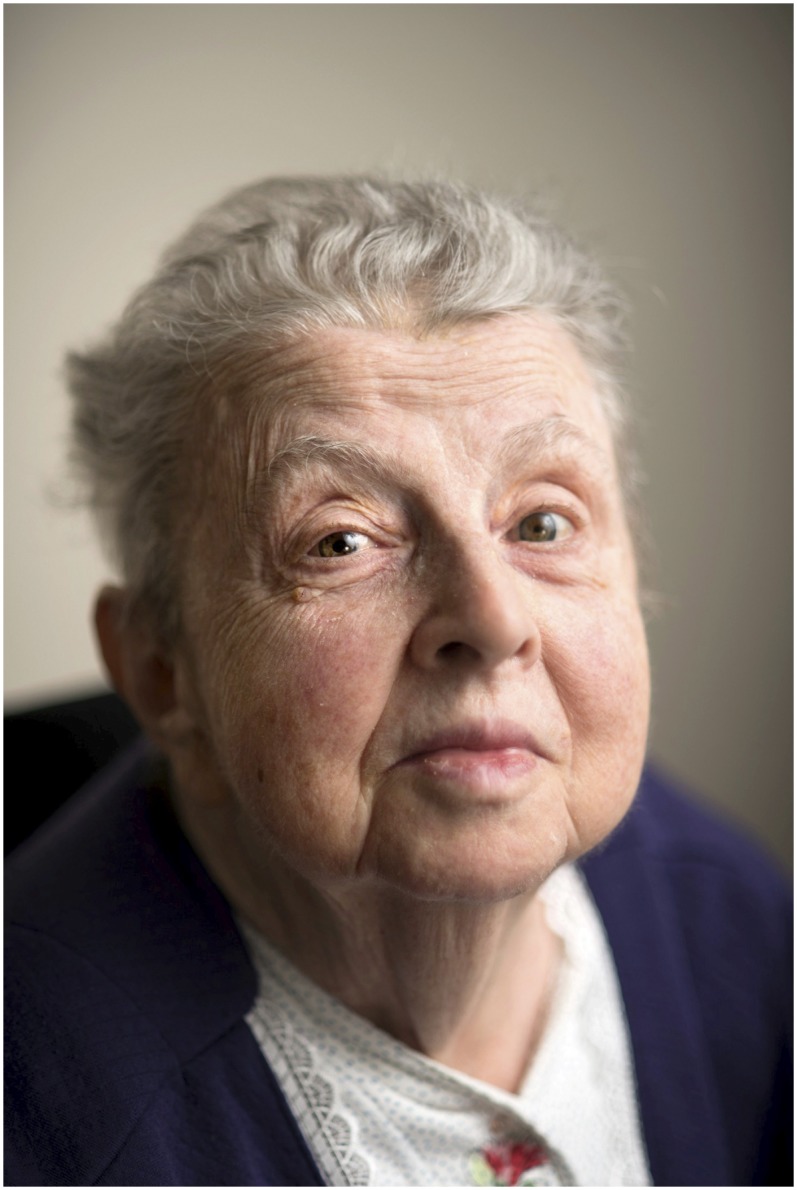
Clara Southmayd Ludlow Medal recipient Ruth Nussenzweig, MD, PhD.

**Figure:**
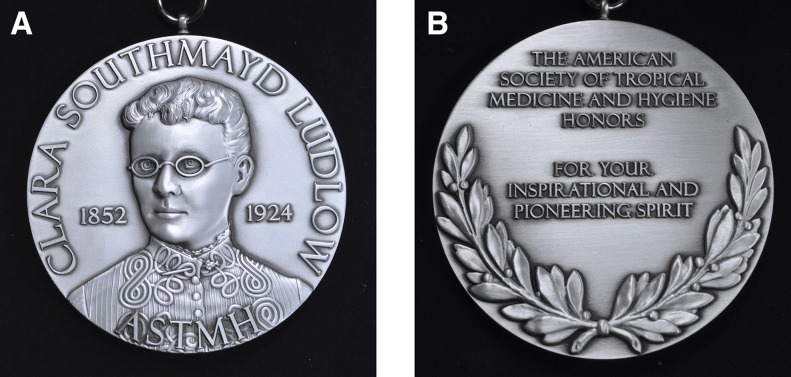
Front (A) and back (B) of the Clara Southmayd Ludlow Medal.

Since its beginnings in 1903, the American Society of Tropical Medicine and Hygiene (ASTMH) has been deeply committed to pursuing scientific excellence in tropical medicine, hygiene and, to use today’s term, global health. This commitment has been expressed in part through recognizing individuals who have made significant contributions to the field. In the deeply held traditions of academia, recognition is provided by awarding medals that are presented in the Awards Ceremony that opens the international Annual Meeting of the ASTMH. In the presence of colleagues and family, honored individuals ascend the stage and accept commemorative medals named for giants in tropical medicine: Bailey K. Ashford, Ben Kean, Joseph Augustin LePrince, Donald Mackay, and Walter Reed.

Long absent has been a female “giant” in tropical medicine. In 2015, the ASTMH Presidents and Council sought to address this omission. A special taskforce of 15 members, chaired by then President-elect Patricia Walker, representing both elected and appointed leaders diverse in geography, expertise, and gender, offered the Council a recommendation for action, approved by the Council in 2016, that the Society create a medal named for an iconic female leader in tropical medicine. Then-President Stephen Higgs announced the creation of this new medal during the Awards Ceremony of the 2016 Annual Meeting.^1^ President Patricia Walker solicited a call for nominations, resulting in 150 submissions naming 57 outstanding candidates as either potential recipients of the medal or after whom the medal should be named. The taskforce enthusiastically embraced the exacting task of selecting a female leader whose name and image would be cast into the new medal and also proposed its first recipient. At its 2017 midyear meeting, the Council unanimously accepted recommendations for the woman whose contributions will be forever recognized by this medal, and its first awardee, whose contributions are emblematic of the pioneering and inspiring spirit of the medal’s namesake.

The medal is named for Clara Southmayd Ludlow, PhD. We would like to think that she had a sense of the history that she was making in 1908 as our first woman^2^ and first nonphysician member. Comprehensive biographies published by Kitzmiller and Ward in 1987^3^ and Carpenter in 2005^4^ provide us with much information. Clara Ludlow was born in 1852 in Pennsylvania. Her mother was Anna Mary (nee Hunt) Ludlow and her father, Jacob Rapelyea Ludlow, was a medical officer in the U.S. Civil War and at the time of her birth, a well-established physician. One brother, David Hunt Ludlow, become a physician, whereas another brother, Henry Hunt Ludlow, pursued a military career that resulted in a posting to the Philippines and a rank of Colonel. Dr. Ludlow’s early passion was music. She graduated in 1879 from the New England Conservatory of Music. However, in the 1880s, her interests turned toward science. She attended Mississippi Agricultural and Mechanical College (MAMC) in Starkville, Mississippi, from 1897 to 1900. It was here that she began to formulate an interest in mosquitoes under the tutelage of George W. Herrick, a professor of biology. Ludlow graduated with a Bachelor of Science in Agriculture from MAMC in 1900 at the age of 48, and she earned a Master of Arts in Botany from Mississippi A&M in 1901.

Ludlow’s archived works reveal that after graduation she traveled to Manila, Philippines, to visit her brother Col. Henry Hunt Ludlow stationed with the U.S. Army. It was during this time in Manila that her fascination with military medicine began. Ludlow’s studies of disease-bearing mosquitoes contributed greatly to the well-being of those stationed at the U.S. Army posts around the world. In 1904, she was a lecturer on mosquitoes and disease at the Army Medical Museum in Washington, D.C. Interestingly, she corresponded with William Gorgas while he was working in the Panama Canal zone^3^ (5 years later, Gorgas was to become ASTMH’s fourth president). In 1907, Ludlow was a Demonstrator of Histology and Embryology at George Washington University, and in 1908, she received her Doctor of Philosophy from that university. Her dissertation was entitled *The Mosquitoes of the Philippine Islands: The Distribution of Certain Species and Their Occurrence in Relation to the Incidence of Certain Diseases.* Her expertise, commitment, and strength of character are to be admired and celebrated given the significant barriers women faced socially and in the male-dominated scientific community. Given this, it is remarkable that Ludlow was appointed as an Anatomist at the Army Medical Museum, and in 1921, referred to herself as Entomologist in charge of the Entomology Department at the Museum. An article in the November 20, 1924, *Sioux City Journal* (Iowa) suggests that Ludlow received little support from her physician father. The article states, “Her education was obtained under inevitable difficulties presented by the roving life of an army child and the equally inevitable opposition of her father, who frequently and fervently assured her that her thirst for knowledge was positively unladylike.”

In her lifetime Ludlow published 53 papers, the titles of which are listed in a previous publication.^3^ She died of cancer in 1924, and as a final display of her status and contributions, she was buried in Arlington National Cemetery.

The first recipient of the Clara Southmayd Ludlow Medal is Ruth S. Nussenzweig, MD, PhD, whose extraordinary contributions forever changed malaria vaccine research. At a time when it was thought that a malaria vaccine was impossible, her work (with husband/collaborator Victor Nussenzweig) showed otherwise, paving the way for today’s malaria vaccine efforts. She has been described as focused, creative, and with a powerful and indomitable personality. Among her many recognitions, she is a member of the National Academy of Sciences, and in 1997, she was the first female recipient of the ASTMH Joseph Augustine LePrince Medal. For much of her career, Dr. Nussensweig was an active ASTMH member, publishing over 250 papers during a 50 year period, with 24 papers published in the *American Journal of Tropical Medicine and Hygiene*.

Going forward, the Society will annually solicit nominations for the Ludlow Medal, which will represent success despite obstacles to advance the field of tropical medicine. Dr. Clara Southmayd Ludlow was remarkable, talented, unafraid, and unapologetic for her work. Recipients of this medal will be individuals—male or female—who persist despite the odds, are pioneering and inspirational, and whose work contributes to understanding and advancement of the field of tropical medicine.
